# Single-cell stable isotope probing in microbial ecology

**DOI:** 10.1038/s43705-022-00142-3

**Published:** 2022-07-06

**Authors:** Uria Alcolombri, Roberto Pioli, Roman Stocker, David Berry

**Affiliations:** 1 Institute of Environmental Engineering, Department of Civil, Environmental and Geomatic Engineering, ETH Zurich, Zurich, Switzerland; 2 Division of Microbial Ecology, Department of Microbiology and Ecosystem Science, Centre for Microbiology and Environmental Systems Science, University of Vienna, Vienna, Austria

**Keywords:** Environmental microbiology, Stable isotope analysis

## Abstract

Environmental and host-associated microbiomes are typically diverse assemblages of organisms performing myriad activities and engaging in a network of interactions that play out in spatially structured contexts. As the sum of these activities and interactions give rise to overall microbiome function, with important consequences for environmental processes and human health, elucidating specific microbial activities within complex communities is a pressing challenge. Single-cell stable isotope probing (SC-SIP) encompasses multiple techniques that typically utilize Raman microspectroscopy or nanoscale secondary ion mass spectrometry (NanoSIMS) to enable spatially resolved tracking of isotope tracers in cells, cellular components, and metabolites. SC-SIP techniques are uniquely suited for illuminating single-cell activities in microbial communities and for testing hypotheses about cellular functions generated for example from meta-omics datasets. Here, we illustrate the insights enabled by SC-SIP techniques by reviewing selected applications in microbiology and offer a perspective on their potential for future research.

## Introduction

The field of microbial ecology has been transformed by the development of sequencing methods and meta-omics analyses, as their application has provided an unprecedented view of the diversity and function of environmental and host-associated microbiomes. While these tools have been valuable for generating hypotheses about microbial interactions and microbiome function, alternative methods are often needed for testing such hypotheses. Stable isotope probing (SIP) is a powerful approach for experimentally identifying specific metabolisms within a microbiome, allowing researchers to interrogate these complex microbial communities [1]. Microbial communities can be probed with stable isotopes by a variety of means, such as evaluating the isotope content of phospholipid fatty acids separated with liquid chromatography [2, 3], or by sequencing of isotope-labeled DNA or RNA that has been separated from unlabeled DNA or RNA by density centrifugation [4, 5]. These are valuable approaches for bulk analysis of microbial communities, and all have their strengths and weaknesses (Table 1 and references therein). However, one common drawback of all of these techniques is that they do not capture spatial information and inter-cell variation in activity. Single-cell SIP (SC-SIP) techniques are particularly well suited to the study of physiological diversity among cells in a community and of spatial structuring within communities: SC-SIP approaches can resolve isotope abundances on a fine spatial scale, are compatible with complementary imaging approaches, and in some cases can even be used to identify individual cells on the basis of their function for sorting and further analysis. Combined with meta-omics data and process measurements, SC-SIP can thus facilitate research bridging scales from single-cell activity to community- and ecosystem-level processes.

**Table 1:** Key technologies. Summary of the key technologies used in SIP, and their main features and pros and cons.

Technology class	Main features	Pros	Cons	References
Single-cell/spatially explicit methods				
Raman microspectroscopy	Measurement of molecular bonds and heavy isotopes in molecules.	Non-destructive measurement (i.e., compatible with live-cell imaging and post-measurement analysis). Minimal sample preparation.	Possible interference from autofluorescence. Chemical fingerprint rather than exact chemical composition is determined. Isotopes of nitrogen are harder to detect.	[12]
Infrared microspectroscopy (IR)	Measurement of molecular bonds and heavy isotopes in molecules. For differences between Raman and IR, see [93].			[42, 93]
Nanoscale-resolution secondary ion mass spectrometry (NanoSIMS)	Nanometer-scale resolution measurement of elements and isotopes.	High mass resolution. High spatial sensitivity. Compatible with isotopes of H, C, N, P, S, Fe, and others.	Destructive. Suitable only with dry samples.	[14, 94]
Imaging mass spectrometry	Spatial metabolomics with a resolution on the order of tens of micrometers. Destructive or non-destructive measurement, depending on the method.	High mass resolution. Detects metabolites and not just elements or chemical bonds.	2D-imaging. Relatively low spatial resolution.	[95]
Bulk community analysis methods				
Isotope ratio mass spectrometry	Measurement of isotope content, either in bulk biomass or preceded by GC or HPLC separation.	Useful to identify active members of a community. Easy to deploy in the environment. The basis of all SC-SIP.	Destructive. Blind to single-cell variation. Bulk analysis, does not provide spatially resolved information	[95, 96]
PLFA-SIP	Measurement of ^13^C content in individual phospholipid fatty acids as biomarkers for specific microbial groups			[2]
DNA-SIP	Separation of heavy-isotope-labeled DNA from unlabeled DNA by density-gradient centrifugation followed by sequencing of fractions of different densities.			[4]
RNA-SIP	Separation of heavy-isotope-labeled RNA from unlabeled RNA by density-gradient centrifugation followed by sequencing of fractions of different densities.			[5]
CHIP-SIP	Analysis of isotope incorporation into rRNA. rRNA is extracted from samples, hybridized to a taxon-specific microarray and measured with NanoSIMS.			[97]
Protein-SIP	SIP followed by metaproteomics. Measurement of isotope incorporation into peptides.			[7]
SIP metabolomics	SIP followed by metabolomics. Measurement of isotope incorporation into metabolites.			[98, 99]
Complementary methods				
Microfluidics	Manipulation of fluids and chemical conditions at the microscale.	Enables short- and long-term single-cell imaging under controlled conditions Allows cell sorting.	Small cell numbers analyzed. Challenging post-measurement analysis.	[12, 100, 101]
Fluorescence in situ hybridization	Fluorescent (or elemental) labeling of individual cells using molecular probes for identification of microbes.	In combination with SC-SIP, it provides a direct link between phenotype, genotype and spatial arrangement.	Destructive.	[102]

**Fig. 1:**
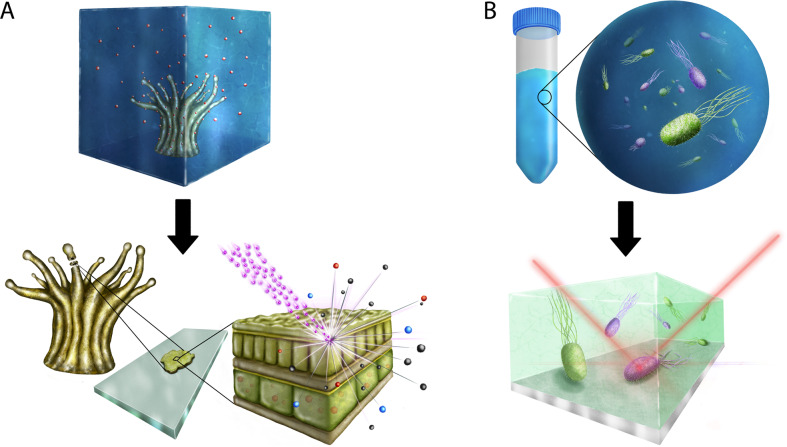
Single-cell stable isotope probing in microbial ecology. Single-cell isotope probing encompasses a range of techniques. Shown are schematics of two main techniques with example applications. **A** NanoSIMS applied to a ^13^C/^15^N isotopically labeled coral to determine nutrient allocation and cross-feeding within the coral holobiont. **B** Raman microspectroscopy applied to identify phenotypic heterogeneity within a clonal bacterial community. In (**A**) (top), a coral or a coral polyp is incubated with an isotope of interest, as indicated by red speckles. (Bottom) A thin chemically-fixed coral section is analyzed by NanoSIMS to obtain its isotopic composition by bombarding its surface with a focused primary ion beam (pink speckles) and by measuring the masses of ejected secondary ions (red, blue and black speckles). In (**B**), (top) a clonal bacterial population is heterogeneously tagged by incorporation of different levels of labeled substrate (pink highly labeled, green unlabeled/partially labeled). (Bottom) Using a confocal Raman microspectroscope, the inelastic scattering of photons from the sample, also known as Raman scattering, is detected from a single cell exposed to a focused laser beam (red line). The recorded Raman scattering is then analyzed to obtain the level of isotope incorporation from a single cell (e.g., D_2_O incorporation, as measured by the presence of C–D bonds). In most cases, the sample preparation and measurement process in Raman microspectroscopy are non-destructive and cells are measured in a solution that does not interfere with the analysis.

SC-SIP has to date been successfully employed to study diverse questions in microbial ecology. These include studies of cellular activities and metabolic pathways, intercellular physiological heterogeneity, and spatial structuring of cellular activities in populations and communities. SC-SIP has already been applied to a wide range of sample types including pure cultures, co-cultures and defined communities, host–microbe systems (e.g., mice and their gut microbiota, corals and their photosynthetic symbionts, sponges and their high and low microbial diversity symbiont communities, and amoebae and their chlamydial symbionts), and samples from a wide range of environments (e.g., freshwater, marine, wastewater, gut). Here, we review a number of elegant examples in which SC-SIP has enabled fundamental insights into cell–cell interactions including symbiosis, cross-feeding, syntrophy, infection, and phage–bacteria interactions (selected examples are illustrated in Fig. 1).

SIP most commonly employs stable isotopes of hydrogen, nitrogen, and carbon, either individually or in combination. The heavy isotope of hydrogen - deuterium - can be used in the form of heavy water (D_2_O) or organic-D [6]. The fate of nitrogen can be followed using ^15^N-labeled nitrogenous compounds such as dinitrogen gas, ammonium, or organic N (e.g., amino acids) [7]. To probe carbon metabolism, ^13^C can be administered as a tracer in the form of CO_2_ (or in aqueous phase as bicarbonate) or organic-C [1, 4, 8, 9]. Alternatively, whole cells can be pre-labeled with heavy isotopes prior to an experiment. This can be done either by employing a pulse-chase design (i.e., first labeling the cells in a microbial community and then removing the label and further incubating the community to evaluate subsequent transfer of label to other cells), or by addition of pre-labeled cells to a microbial community before incubation [10]. Whole cell pre-labeling provides an approach to study processes such as predation, necrotrophy, and saprotrophy by measuring transfer of isotopes from pre-labeled prey cells to predator cells, and can also be used as a means to quantify cellular activity via the measurement of heavy isotope dilution rather than accumulation [10, 11]. The diversity of labeling strategies, and the availability of multiple isotopes that can be used simultaneously, allows for a broad range of creative and sophisticated experimental designs.

Excellent reviews have been published on the main technologies and instrumentation used in SC-SIP (e.g., [12–14]), which we refer the reader to for details about these technologies (see also Key Technologies Box). The aim of this mini-review is to provide an illustrative overview of how SC-SIP has been applied to advance important research questions in microbiology and to offer a perspective on future directions and opportunities. The selected examples are not intended to provide comprehensive coverage, but rather to highlight the types of biological questions that SC-SIP can answer and to demonstrate key experimental setups.

## Vignettes—what have we learned?

### Cellular metabolism

In many microbial ecosystems, the detection of a cell is no guarantee that the cell is metabolically active or even viable. Microbial dormancy, or existence in a persister state, is important in chronic infectious diseases as well as in the environment, where it contributes to “microbial seed banks” [15]. In fact, dormancy has been speculated to be the predominant state of microbes in some environments [16]. It is therefore important to have tools to assess the viability and metabolic activity of environmental microbes. Detection of DNA is insufficient for this purpose, as extracellular DNA can be extremely stable [17]. Use of rRNA as an activity marker is problematic due to dramatic differences in rRNA stability in different organisms [18]. Stains such as propidium monoazide and propidium iodide that can be used to indicate cells with compromised cell membranes [19, 20] all suffer from divergent protocols for different microbes [21], thereby limiting their applicability in diverse microbial communities. Some newer methods, such as fluorescence-based biorthogonal non-canonical amino acid tagging (BONCAT) and D-amino acid labeling of peptidoglycan [22, 23], are promising alternatives for the detection of single-cell activity. However, SIP remains uniquely suited for the identification of microbial activity without necessitating the addition of a chemically modified compound or fluorescent dye that might interfere with cellular physiology.

SC-SIP has been successfully used to detect cellular microbial activity in host-pathogen systems. A series of studies applying SC-SIP have provided clear insights into the growth of pathogenic bacteria in cystic fibrosis (CF). CF is a genetic disease that results in the buildup of mucus in the respiratory tract, and, as a consequence, to chronic lung infections caused by pathogens such as *Staphylococcus aureus* and *Pseudomonas aeruginosa*. As these are long-term infections, the level of cellular activity of these biofilm-forming pathogens has remained an open question. In order to evaluate the cellular activity of *S. aureus* and *P. aeruginosa* in cystic fibrosis biofilms, Kopf et al. [24] quantified the incorporation of deuterium from heavy water and ^15^N from ^15^N-ammonium into microbial biomass using chemostat reactors operated to produce different bacterial growth rates. They demonstrated that the growth rate can be inferred from cellular deuterium incorporation, and were able to use the dual-labeling strategy to estimate the relative contributions to bacterial growth of ammonium and amino acid assimilation. By then applying heavy water to freshly expectorated sputum from CF patients, they found that the growth rate of *S. aureus* was at least two orders of magnitude lower than that typically obtained when cells are grown in the laboratory, and that there was considerable cell-to-cell heterogeneity in growth rate in the *S. aureus* population [25]. It thus appears that the in vivo physiological state of *S. aureus* differs considerably from that under standard laboratory growth conditions. This finding has important implications for the design of effective CF treatment strategies, for example because physiological state and growth rate can dramatically alter bacterial resistance to antimicrobials. The approach also has potential clinical applications. Neubauer et al. [26] measured deuterium incorporation into fatty acids isolated from sputum samples using gas chromatography-mass spectrometry and found that deuterium incorporation into two microbially-produced branched-chain fatty acids (anteiso-C15:0 and anteiso-C17:0) was a robust proxy for pathogen growth in CF sputum. Since these measures can be performed in high-throughput, this discovery raises the possibility of rapid screening of clinical samples to determine the extent of pathogen growth.

Microbial dormancy has been explored in bacteria and protozoa using SC-SIP in a variety of settings. Chlamydia have a biphasic lifestyle in which they are either actively replicating within host cells or in a non-replicative infectious extracellular state. To evaluate whether the extracellular cells are in fact metabolically quiescent, extracellular chlamydia were incubated with ^13^C-labeled phenylalanine and uptake of the labeled amino acid was detected by Raman microspectroscopy [27]. The observation that extracellular chlamydia were capable of amino acid uptake and protein synthesis overturned a long-held belief that extracellular cells (also known as “elementary bodies”) exist in a spore-like dormant state during transmission between hosts. The in situ metabolic state of the parasite *Leishmania mexicana*, which forms granulomatous lesions in its hosts, was investigated with heavy water SIP combined with imaging mass spectrometry. Imaging of granulomas in infected mice revealed a mixed population of active and non-active *Leishmania* cells within the granulomas, and, surprisingly, also a large number of metabolically quiescent cells in the surrounding mesothelium [28], which the authors speculated could be an important mechanism by which the pathogens survive drug treatment. The properties of bacterial spores have been studied using SC-SIP. The water permeability of the spore coat of *Bacillus subtilis* was evaluated using a heavy water tracer and Raman microspectroscopy [29], and the germination kinetics of spores was quantified at the single-cell level using Raman microspectroscopy and heavy water as well as by measuring changes in the level of the spore component calcium dipicolinate [29–31].

Another example of the use of Raman microspectroscopy to examine cellular activity under different spatial structuring in microbial communities comes from a study of the degradation of hyphae of the fungus *Mucor fragilis* by the soil bacterium *Bacillus subtilis* [11]. Metabolic activity of the bacteria was studied using a combination of ^13^C- and D_2_O-based labeling strategies within a transparent soil microcosm containing *M. fragilis*. The results revealed that both hyphae-attached and planktonic *B. subtilis* are metabolically active under constant hydrated conditions, but that attached bacteria are more metabolically active under wetting–drying cycles typical of soil ecosystems. This indicates that surface attachment may be selected by fluctuating environmental conditions, and demonstrates how ecological hypotheses about spatially explicit microbial activities are amenable to testing by SC-SIP within suitable microcosm setups.

The genomes of microorganisms generally encode a variety of metabolic pathways that can alternately be expressed or repressed according to environmental conditions. In ecological terms, an organism’s fundamental niche, or the number of different pathways at its disposal, may be much broader than its realized niche, or the metabolism that it is employing at any given moment [32, 33]. Methods such as metatranscriptomics and metaproteomics can offer some insights into metabolic activities, but suffer from limitations such as variable RNA and protein extraction efficiencies, differences in the stability and turnover time of target molecules, and the fact that many genes have no known function or are mis- or under-annotated [34]. SIP is a useful tool in identifying specific metabolisms under specific conditions in complex microbial communities, as it does not require prior knowledge of an organism’s metabolic pathways [1, 35].

One example of a metabolic strategy that has been studied with SC-SIP is mixotrophy, the simultaneous use of different sources of energy and carbon. Mixotrophy was quantified in the phagotrophic alga *Ochromonas* sp. strain BG-1 by growing the alga in the presence of either heat-killed ^13^C/^15^N-labeled bacteria or unlabeled heat-killed bacteria and ^13^C-bicarbonate and ^15^N-ammonium [36]. NanoSIMS analysis, as well as bulk isotope-ratio mass spectrometry (IRMS), indicated that *Ochromonas* obtained most of its C (84–99%) and N (88–95%) from consumed bacteria, and that autotrophic activity was detectable but insufficient to support population growth. Detection of chemoautotrophy and heterotrophy in complex microbial communities has also been enabled by SC-SIP. Using marine water samples incubated with ^13^C-bicarbonate and ^15^N-amino acids followed by NanoSIMS measurement, Dekas and coworkers [37] found that only a minority (4–17%) of the active cells in the samples were chemoautotrophic. When antibiotics were added to the samples to specifically inhibit bacteria, the fraction of active cells with chemoautotrophic metabolism increased. This suggested that archaea, whose activity was not inhibited by the antibiotic, might have been responsible for most of the chemoautotrophy. To confirm this hypothesis, the authors used fluorescent in situ hybridization (FISH) to fluorescently stain Thaumarchaeota as well as bacteria and evaluated FISH-stained cells using NanoSIMS, finding that indeed this group of archaea displayed chemoautotrophic activity whereas bacteria did not. A similar pattern of bacterial heterotrophy and archaeal autotrophy was found in anoxic marine sediments using heavy water and ^13^C bicarbonate labeling and fatty acid analysis [38]. By going a step further to identify the microbes involved, SC-SIP can provide a tool for the discovery of novel microbes performing a specific function. For example, to identify autotrophs in marine samples, Jing and coworkers [39] incubated samples with ^13^C-bicarbonate and then sorted ^13^C-labeled cells using Raman activated cell ejection (RACE). By performing shotgun sequencing on the sorted cells, they were able to reconstruct nearly complete genomes from novel, uncultured *Synechococcus* spp. and *Pelagibacter* spp., which confirmed that they encoded genes for photoautotrophy, and as they had incorporated ^13^C-bicarbonate in the incubation were active as autotrophs under these conditions. Further, SC-SIP has been employed to distinguish between different organic carbon utilization pathways. By growing pure cultures of a *Pseudomonas* sp. or *Escherichia coli* in the presence of heavy water and either ^13^C-glucose or ^13^C-naphthalene, Xu et al. [40] observed with Raman microspectroscopy differences in synthesized phenylalanine deuteration between the two organisms, which they proposed was due to differences in their phenylalanine biosynthesis pathway.

The above examples illustrate how SC-SIP enables monitoring of microbial cellular activities and identification of metabolic processes in complex environments, in a spatially and temporally resolved manner. We expect that recent developments in non-destructive approaches such as femtosecond stimulated Raman spectroscopic imaging [41] and high resolution optical infrared microspectroscopy [42] will facilitate further applications of SC-SIP to discover new single-cell microbial activities in the environment.

### Phenotypic heterogeneity

While microbial communities are composed of genetically diverse assemblies of organisms, biological diversity can also be found at a lower level of organization in the form of phenotypic heterogeneity [43]. Phenotypic heterogeneity is a widespread phenomenon in microbial ecology [44–53]. The sources of this heterogeneity range from ecological factors and cell-specific dynamics to molecular noise in gene expression that is transmitted to metabolic processes [54]. Phenotypic heterogeneity can provide microbial populations with several ecological advantages [55]. For example, it allows the population to adopt a bet-hedging strategy, which enables part of the population to survive unfavorable conditions (e.g., antibiotic treatment) [43, 56]. Phenotypic heterogeneity within microbial populations can be observed in a variety of settings, even in a constant, homogenous environment [57]. Accounting for phenotypic heterogeneity is thus important in many processes in microbial ecology, with relevance in both environmental processes and healthcare.

During the last decade, evidence that phenotypic heterogeneity plays an important role in the environment has begun to accumulate, to a large extent based on single-cell technologies, and in particular SC-SIP. For example, Schreiber et al. used NanoSIMS to measure the metabolic activities of the bacterium *Klebsiella oxytoca* under conditions of mixed substrate availability and substrate shifts [52]. *K. oxytoca* is a N_2_-fixing bacterium, yet will preferentially take up NH_4_ when present. The authors showed that limiting amounts of NH_4_ induced a heterogeneous nitrogen acquisition strategy in *K. oxytoca*, where part of the population acquired nitrogen through N_2_ fixation and the remainder used NH_4_. Furthermore, the level of within-population heterogeneity in N_2_ fixation increased with increasing NH_4_ supply. They further showed that the rate of single-cell N_2_ fixation during NH_4_ limitation correlated positively with the cells’ ability to grow after shifting the culture to NH_4_-depleted conditions. They therefore established that phenotypic heterogeneity is an effective solution to both nutrient limitation and fluctuations, two important ecological challenges that many microorganisms encounter in their natural habitat. In an environmental study, Zimmermann and coworkers describe phenotypic heterogeneity in the green sulfur bacterium *Chlorobium phaeobacteroides*, by examining N_2_ and CO_2_ fixation by single cells from the Cadagno lake in Switzerland [58]. The researchers incubated lake water with ^15^N_2_ and ^13^CO_2_ in the presence or absence of NH_4_ and used cell sorting based on the auto-fluorescence of *C. phaeobacteroides* to concentrate the cells from the environment by two orders of magnitude. Using NanoSIMS they then measured the incorporation of the stable isotopes and found that *C. phaeobacteroides* fixes N_2_ only in the absence of NH_4_. Additionally, they were able to show that N_2_ and CO_2_ fixation is heterogeneous and positively correlated across cells, suggesting that the fixation of N_2_ and the fixation of CO_2_ interact and positively facilitate one another within individual cells. In subsequent work, Zimmermann and coworkers used again *C. phaeobacteroides*, yet this time limiting their cultures for an H_2_S electron donor [59]. They showed that despite the different limitation modes, phenotypic heterogeneity still emerged, demonstrating that nutrient limitation is a general driver of phenotypic heterogeneity in microbial populations.

An example of phenotypic heterogeneity in heterotrophic microorganisms has been demonstrated by Sheik and coworkers, who investigated *in situ* substrate assimilation by single cells of the wastewater filamentous bacterium *Candidatus Microthrix parvicella* at different temperatures and in response to alternating aerobic–anoxic conditions [60]. Using NanoSIMS, they found that ^13^C-oleic acid and ^13^C-glycerol-3-phosphate assimilation occurred under the different conditions in only 21–55% of the *Ca. M. parvicella* cells, whereas the remainder of cells did not exhibit any substrate assimilation despite being intact and alive. The authors suggested that the phenotypic heterogeneity of *Ca. M. parvicella* cells enables them to rapidly adapt to the fluctuating environmental conditions prevalent in wastewater treatment plants, such as alternating oxygen availability and temperature variations. Interestingly, there was marked phenotypic heterogeneity also among individual *Ca. M. parvicella* cells belonging to the same filament, despite them experiencing a very similar environment and having identical genetic backgrounds. In another study of phenotypic heterogeneity within heterotrophic microbial populations, the variation in sugar metabolism has been found using SC-SIP within clonal bacterial populations of *E. coli*. By culturing *E. coli* in chemostats in the presence of ^2^H/^13^C-labeled sugars, Nikolic et al. [53] found large variation in metabolic activities among single cells, both in overall assimilation rates and in sugar-specific assimilation, as measured by NanoSIMS. The authors proposed that this heterogeneity could be at least partially explained by variation among cells in gene expression, or phenotypic heterogeneity at the transcriptional level.

The examples provided above illustrate how SC-SIP methods can be used to determine the prevalence and relevance of phenotypic heterogeneity in different microbial environments. Phenotypic heterogeneity has been shown to be an effective solution to both nutrient limitation and environmental fluctuations commonly found in many microbial habitats. We anticipate that SC-SIP techniques will continue to be at the forefront of studying microbial phenotypic heterogeneity.

### Symbiosis and cross-feeding

Interactions among symbiotic microbes are mediated on a molecular level and are manifested in different ways depending on the physiology of the participants [61]. Understanding the nature and the underlying molecular mechanism of a symbiotic interaction can therefore be a complex task. Specifically designed to monitor the metabolic exchange that binds organisms together in symbiosis, SC-SIP has aided in the understanding of symbiotic relationships between microbes [62]. In particular, SC-SIP has been widely used to define the currencies sustaining the interaction between symbiotic partners. Negative interactions have also been revealed using SC-SIP analyses. For example, viral infection of *Emiliania huxleyi*, *Synechococcus* and *E. coli* have been visualized using NanoSIMS and BONCAT to quantify viral production [63]. In this section we highlight several examples of how SC-SIP has been used to reveal a metabolic interaction between microorganisms and to identify and quantify the currency that is exchanged among symbiotic organisms.

Corals and their symbiotic photosynthetic algae belonging to the genus *Symbiodinium* represent perhaps one of the most famous symbiotic interactions. This mutualistic endosymbiotic relationship is based on the photosynthetically derived carbon provided by the *Symbiodinium* partner to its coral animal host. SC-SIP was used to study the flux of nutrient among the organisms that form the complex holobiont of the coral under a wide range of scenarios, including under normal photosynthesis-driven carbon assimilation [64], heat stress [65] and mixed autotrophic and heterotrophic feeding strategies [66]. In a recent study, SC-SIP was used to quantify the nutrient exchange between the coral *Pocillopora damicornis* and its symbiont *Symbiodinium* in the presence or absence of a bacterial pathogen of corals, *Vibrio coralliilyticus* [67]. The relationship between *P. damicornis* and *V. coralliilyticus* is commensal under homeostatic conditions, but in response to an increase in temperature, *V. coralliilyticus* becomes pathogenic, leading to coral tissue lysis, expulsion of the symbiotic algae, and eventually coral death. Using NanoSIMS after incubating samples in the presence of ^13^C-bicarbonate sea water, the researchers revealed that pathogenic infection of the coral with *V. coralliilyticus* reduced carbon assimilation in *Symbiodinium*, while ^13^C-assimilation by the coral host increased. The authors suggested that a defending coral is likely to have a higher demand for energy, and therefore translocated fixed carbon to sustain the extra energy consumption needed for defense. Using SC-SIP the authors discovered that in addition to the physiological condition of the host, nutritional interactions and carbon allocation between symbiotic partners are key to shaping coral‒symbiont‒bacteria interactions and progression of pathogenicity.

The symbiosis between sponges and bacteria is another prevalent and ecologically important interaction known to play an essential role in the cycling of dissolved organic matter (DOM) in the ocean [68, 69]. Sponges and their symbionts have a variety of nutritional strategies; however, the majority of sponges are heterotrophic filter feeders, which capture their food via specialized cells called choanocytes. The contribution that bacterial symbionts make to sponge heterotrophy has only recently been quantified. In an investigation of this symbiotic relation, SC-SIP was used to compare the consumption of different sources of DOM by sponges with a high or low abundance of microbial symbionts. In an in situ feeding experiment using different types of ^13^C-DOM and ^15^N-DOM, the authors demonstrated that DOM was the primary carbon source for both sponge types, accounting for approximately 90% of their heterotrophic diets. Microbes accounted for the majority (65–87%) of DOM assimilated by the sponges with a high abundance of microbes and ~60% of their total heterotrophic diet, but less than 5% in the sponges containing a low abundance of microbes. Unlike what had been commonly assumed, these findings quantitatively demonstrate that not only the host’s choanocyte cells but also the microbial symbionts are actively, and in proportion to their abundance, involved in DOM processing.

SC-SIP is a powerful tool to study the exchange of metabolites between symbiotic partners. In particular, it can be used to study scenarios in which one of the partners in the symbiosis becomes redundant, for example when an external source of nutrients becomes available. In a recent study, the symbiosis between the marine haptophyte *Braarudosphaera bigelowii* and the cosmopolitan marine cyanobacterium UCYN-A was challenged by externally supplying the dissolved inorganic nitrogen (DIN) that forms the basis of the symbiosis [70, 71]. In much of the ocean, primary production is restricted by the availability of nitrogen and many phytoplankton have adopted strategies to acquire nitrogen [72]. In the case of the symbiosis between *B. bigelowii* and the UCYN-A cyanobacteria, which is usually found in nitrogen-depleted waters, the UCYN-A uses N_2_ fixation to meet a large portion of the haptophyte’s requirements for nitrogen and in return the haptophyte provides photosynthetically fixed carbon. Providing the symbiotic partners with an external source of the stable isotopes ^15^N_2_, ^15^NO_3_^−^, ^15^NH_4_^+^ and H^13^CO_3_, the authors could determine using SC-SIP and CARD-FISH the rates of nitrogen fixation, dissolved inorganic nitrogen uptake, and carbon fixation [70]. The authors found that, although the symbiosis with UCYN-A is based upon N_2_ fixation and sharing with the haptophyte, in nitrogen-replete waters containing DIN the haptophyte still relies on UCYN-A nitrogen fixation for a large proportion of its nitrogen. They propose that despite the availability of external sources of DIN, this biogeochemically important symbiosis could even be maintained in nitrogen-rich coastal waters [70].

SC-SIP methods have also been employed to illustrate how energetic currency in the form of electron transfer can mediate a symbiotic relation, and in particular in energy-limited environments. Methane oxidation with sulfate, which is a process that limits the entry of the greenhouse gas methane into the atmosphere, is carried out at marine methane seeps and involves cross-feeding between anaerobic methanotrophic archaea (ANME) and sulfate-reducing bacteria (SRB) [73]. Using a combination of rate measurements and SC-SIP, the underlying interdependencies within this uncultured symbiotic partnership have been elegantly untangled. The authors revealed the cross-feeding at the basis of this symbiotic partnership by demonstrating that ANME in deep-sea sediments can be catabolically and anabolically decoupled from their syntrophic SRB partners by providing them with an artificial oxidant. Their findings suggest that the syntrophic oxidation of methane is mediated via interspecies extracellular electron transfer [73]. Interestingly, unlike the symbiosis between the UCYN-A and the haptophyte discussed above, by using SC-SIP the authors demonstrated that this partnership was compromised by the addition of external resources, suggesting that some seemingly obligate symbioses are in fact context-dependent.

Investigations using SC-SIP have also made important contributions to identifying the trophic interactions of taxa within microbial food webs, by providing isotopically labeled metabolites and tracking their passage. For example, using heavy-water SIP incubations followed by Raman-activated cell sorting and metagenome sequencing, Pereira and coworkers found indications of cooperative mucosal glycan degradation within members of the family Homothermaceae in the mouse gut microbiota [74]. Specifically, the authors found that different members of the group encoded different enzymes that contribute to step-wise degradation of mucosal glycans, suggesting that degradation is a mutual, community-level process. In many systems, however, the complex passage of labeled metabolites can make it difficult to determine the trophic roles of individual taxa, for example, to distinguish primary degraders from cross feeders in a complex multi-species community. To identify primary degraders, a Flow-SIP approach has been developed that has the ability to significantly reduce cross-feeding in complex microbial communities [75]. Using NanoSIMS with ^13^C-bicarbonate as an activity tracer, the approach was used to study nitrifiers in activated sludge. The researchers showed that by applying fluid flow that washes away leaching compounds from the primary degraders they can significantly reduce cross-feeding, allowing primary consumers to be clearly distinguished from other members of the microbial food web. Additionally, this work provided further evidence that fluid flow, a common and crucial feature of many habitats (e.g., [76–78]), may also reduce the amount of cross-feeding in the environment.

Microorganisms rarely exist in isolation [79]. Therefore, the use of SC-SIP, which enables unprecedented access to the molecular trading between microorganisms in their environment, can only be expected to expand in the future. We expect that as the cost declines and the instruments required to use SC-SIP become more widely available, many more microbial food webs and symbiotic interactions, nowadays mostly predicted from observations of co-occurrence, will be revealed and understood on a molecular level.

## Outlook

While SC-SIP has been used in a wide variety of systems in microbial ecology, we expect many more applications and developments of this technology in the near future. For example, subcellular localization of compounds using NanoSIMS has been used in medical research to identify cellular targets of platinum-containing anti-cancer drugs [80], as well as in environmental microbiology to study magnetosomes in magnetotactic bacteria [81] and the role of fungal nanophase particles in mediating fungal-mineral interactions [82]. Subcellular localization of compounds using NanoSIMS could have many additional applications in microbial ecology, such as the localization of metal isotopes in microbial cells to localize enzymes with metal cofactors such as iron, copper, zinc, or nickel [83], or to quantify protein turnover rates in cells [66, 84].

**Fig. 2:**
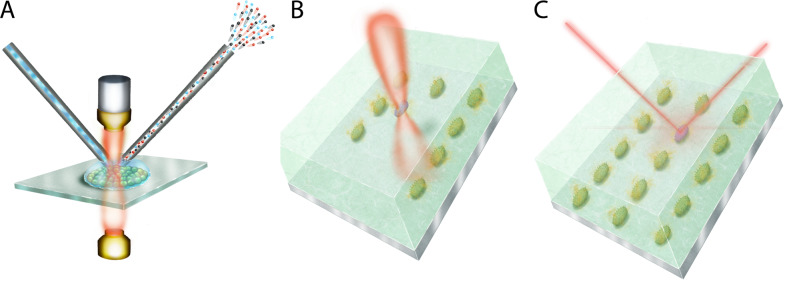
Emerging technologies in single-cell stable isotope probing. The schematics show examples of emerging technologies that will enhance SC-SIP capabilities and provide a wider window into the unseen world of microbes. **A** Multimodal microscopy allows in situ integrative fluorescence imaging, nanospray desorption electrospray ionization mass spectrometry (Nano DESI-MS), and Raman microscopy to study microbial metabolic interactions. A sample is imaged simultaneously with fluorescence microscopy and Raman microscopy to identify a specific cell type or cellular metabolic activity. A nanocapillary is used to inject a mobile phase that collects the exo-metabolites above the sample and sends them through a second capillary to an electrospray ionization mass spectrometer. The signal from all imaging techniques is incorporated into one multimodal image. **B** Multiple optical traps that control the spatial organization of cells in a microfluidic chamber. In the scenario shown, a cell of interest (purple) is placed at the center of a pre-organized array of cells of a different type (green). **C** The cell (purple) can then be inspected using Raman microscopy for its metabolic activity in the context of a specific community composition or community spatial organization. Different cell types or cell organizations can be achieved quickly using multiple parallel laser optical traps to identify each cell type and its location.

The full potential of SC-SIP for future applications in microbial ecology can be unlocked by pairing this technology with technical developments that allow more precise, reliable single-cell interrogation (Fig. 2). This includes for example microfluidic technologies in which cells can be studied under precisely controlled environmental conditions [85, 86], and one or multiple optical traps [87, 88] or single-cell patterning by surface acoustic waves [89], allowing the spatial relationships of cells to be controlled with high precision.

Emerging techniques that show promise include stimulated Raman scattering microscopy, a vibrational spectroscopy technique with rapid and sensitive detection of isotopes in cellular biomass, facilitating measurements over unprecedentedly large areas [90, 91]. Combinations of existing methods, or multimodal imaging approaches such as imaging MS [92], vibrational spectroscopy, and fluorescence labeling/reporters (e.g., transcriptional fusion reporters or BONCAT) [11, 13] have the potential to more comprehensively link microbe identity, activity, and metabolite production in a spatially resolved and non-invasive manner. We fully expect that these and other developments will further increase the scope and power of SC-SIP and fuel exciting new discoveries across microbial ecology.
